# In This Issue: Volume 117, Issue 7, July 2026

**DOI:** 10.1111/cas.70452

**Published:** 2026-07-02

## Super‐Enhancer Formation in Scirrhous Gastric CAFs, and the Presence of a Stromal Field in Non‐Cancerous Tissues

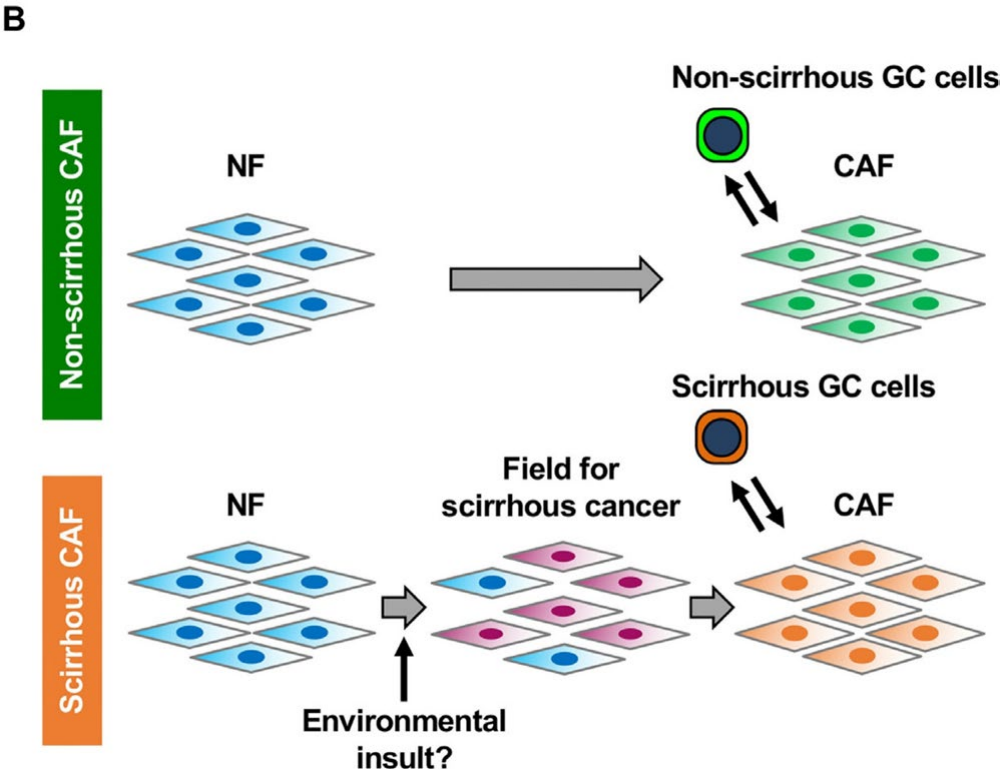

Scirrhous gastric cancer is a rare and aggressive form of stomach cancer in which malignant cells are embedded within a hard and dense extracellular matrix. This matrix is secreted and remodeled by cancer‐associated fibroblasts (CAF), activated stromal cells within the tumor microenvironment that support tumor growth and contribute to treatment resistance.

Researchers have long believed that mutant cancerous cells “educate” normal fibroblasts in stomach tissue and convert them to CAFs. While they are not cancerous by themselves, CAFs undergo epigenetic remodeling, such as histone methylation, to continue supporting cancer cells.

A recent study by Yasukawa et al. examined the specific epigenetic mechanisms by which scirrhous CAFs promote cancer growth. They also studied whether fibroblasts from other non‐cancerous tissues of a patient with scirrhous gastric tumors may be primed to become scirrhous CAFs.

The authors found that the TGFβ1 transcription pathway is highly activated in scirrhous CAFs. This included downstream transcription factors linked to tumor growth, inflammation, and metastasis. Secretions by scirrhous CAFs induced cancer cell migration 2 to 4 times more strongly than non‐scirrhous CAF secretions.

The activation and excessive secretion of these factors is further cemented by epigenetic remodeling in scirrhous CAFs, such as “super‐enhancer” histone acetylation at TGFβ1 target genes. One surprising discovery was that “normal” fibroblasts in people with scirrhous gastric tumors also showed features that enhance cancer cell migration. The authors suspect that some environmental stressors cause these epigenetic changes to occur in the normal stomach and create a microenvironment that promotes scirrhous gastric cancer development.

This discovery also shows that drugs that target “super‐enhancer” histone acetylation could exploit a therapeutic vulnerability in scirrhous gastric cancer. Further research into CAF secretions may reveal additional vulnerabilities and therapeutic targets for the treatment of scirrhous gastric cancer.

https://onlinelibrary.wiley.com/doi/full/10.1111/cas.70408

## Triple‐Mutated HSV‐1 Expressing Soluble B7‐1 Plus CTLA‐4 Blockade Suppresses Lymph Node Metastasis in Tongue Cancer

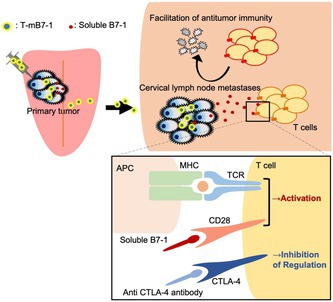

Oral squamous cell carcinoma is one of the most common cancers of the mouth, and its spread to the neck lymph nodes is strongly linked to poor survival. Although immune checkpoint therapies have improved treatment options for some patients, many people still do not respond well or experience severe side effects. Researchers are therefore searching for safer and more effective treatments that can control cancer spread at an earlier stage. One promising approach involves oncolytic viruses, which are specially engineered viruses designed to infect and destroy cancer cells while also activating the body's immune system against tumors.

In this study, researchers utilized a modified herpes simplex virus type 1 (HSV‐1) that produces a soluble form of B7‐1, an immune‐stimulating protein that helps activate T cells. The engineered virus, called T‐mB7‐1, was tested in several mouse models of tongue cancer that closely mimic aggressive oral cancer with rapid lymph node spread. The virus successfully released the immune‐activating protein without losing its cancer‐killing ability. When injected directly into tongue tumors, T‐mB7‐1 reduced tumor growth, suppressed the spread of cancer to cervical lymph nodes, and significantly improved survival. Repeated low‐dose treatments produced especially strong effects, with many mice surviving long‐term. Importantly, combining the virus with cytotoxic T‐lymphocyte‐associated protein 4 (CTLA‐4) blockade, a type of immune checkpoint therapy, further strengthened the anti‐cancer response.

Together, these findings suggest that combining an engineered cancer‐targeting virus with immune checkpoint therapy may offer a powerful new strategy for controlling metastatic oral cancer. The treatment showed strong anti‐tumor effects while remaining safe in preclinical testing. By targeting both cancer cells and the immune environment surrounding metastatic lymph nodes, this approach may help improve outcomes for patients with difficult‐to‐treat oral cancers. Future studies will be needed to confirm safety and effectiveness in humans and to better understand how the treatment activates immune responses against cancer.

https://onlinelibrary.wiley.com/doi/full/10.1111/cas.70410

## Losartan Enhances Radiosensitivity by Reversing Immunosuppressive Tumor Microenvironment Induced by Radiotherapy in TNBC

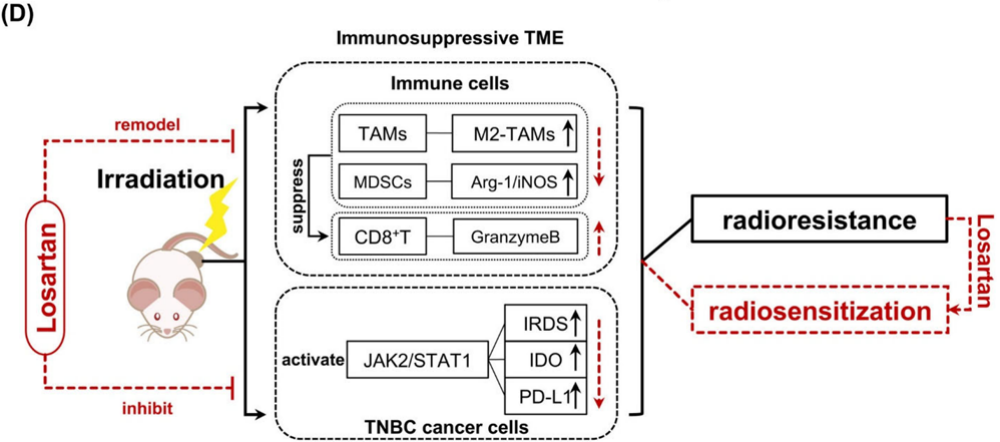

Breast cancer is the most commonly diagnosed cancer among women. Among the breast cancer subtypes, triple‐negative breast cancer (TNBC) is associated with poor prognosis. Currently, TNBC is managed through surgery, chemotherapy, and radiotherapy. Response to radiotherapy is limited in some patients. This is because radiotherapy can suppress immune responses (immunosuppression) in the tumor environment [called the tumor microenvironment (TME)] or induce gene mutations, promoting resistance to radiotherapy (radioresistance). Radiotherapy's efficacy can be enhanced by overcoming this immunosuppression.

Targeting the renin‐angiotensin system (RAS), a hormonal system that regulates blood pressure, fluid balance, and electrolytes, can potentially achieve this goal. The RAS promotes breast cancer growth by maintaining the immunosuppressive state of the TME. As RAS‐related proteins are expressed in both immune cells and cancer cells, RAS inhibitors can prevent cancer growth by targeting immune cells. One such RAS inhibitor is losartan, which specifically inhibits the RAS component angiotensin II receptor 1 (AGTR1). Losartan is known to increase the sensitivity of various cancer cells to radiotherapy.

This study examined if losartan can similarly increase the radiosensitivity of TNBC. In humans, high AGTR1 levels are associated with poor survival only in the TNBC subtype. Thus, mice were artificially implanted with TNBC cells (which multiplied to form a tumor) and treated with radiotherapy (12 Gy) and losartan (dose: 20 mg/kg body weight/day). Losartan potentiated the ability of radiotherapy to suppress tumor growth and increase tumor cell death. Experiments were also performed with TNBC cells, which were pre‐treated with losartan (200 nM) and exposed to radiation. Notably, losartan did not increase the radiosensitivity of TNBC cells but increased the radiosensitivity of the implanted tumor in mice, indicating indirect effects.

Focusing on these indirect effects revealed that losartan altered the abundance of specific immune cells called tumor‐associated macrophages (TAMs). Specifically, the losartan + radiotherapy combination increased the proinflammatory TAMs (M1 macrophages) and decreased the anti‐inflammatory TAMs (M2 macrophages).

The TME also comprises T cells, which are immune cells that directly kill tumor cells. Losartan reactivated the activity of T cells suppressed by radiotherapy. At the molecular level, losartan worked by decreasing the levels of radioresistance‐related (interferon‐related DNA damage resistant signature) and immunosuppression‐related factors (programmed death‐ligand 1 and indoleamine‐2,3‐dioxygenase) and inhibiting the JAK2/STAT1 signaling pathway (involved in regulating immune response and cell death).

Based on these findings, a novel strategy can be developed to treat TNBC using the radiotherapy + losartan combination.

https://onlinelibrary.wiley.com/doi/full/10.1111/cas.70407

